# Optimizing and accelerating the assignation of lineages in *Mycobacterium tuberculosis* using novel alternative single-tube assays

**DOI:** 10.1371/journal.pone.0186956

**Published:** 2017-11-01

**Authors:** María Carcelén, Estefanía Abascal, Marta Herranz, Sheila Santantón, Roberto Zenteno, María Jesús Ruiz Serrano, Emilio Bouza, Laura Pérez-Lago, Darío García-de-Viedma

**Affiliations:** 1 Servicio de Microbiología Clínica y Enfermedades Infecciosas, Hospital General Universitario Gregorio Marañón, Madrid, Spain; 2 Instituto de Investigación Sanitaria Gregorio Marañón, Madrid, Spain; 3 CIBER Enfermedades respiratorias, CIBERES, Spain; 4 Instituto de Salud Pública, Universidad Veracruzana, Jalapa, Veracruz, Mexico; 5 Departamento de Medicina, Facultad de Medicina, Universidad Complutense de Madrid, Madrid, Spain; Chang Gung University, TAIWAN

## Abstract

The assignation of lineages in *Mycobacterium tuberculosis* (MTB) provides valuable information for evolutionary and phylogeographic studies and makes for more accurate knowledge of the distribution of this pathogen worldwide. Differences in virulence have also been found for certain lineages. MTB isolates were initially assigned to lineages based on data obtained from genotyping techniques, such as spoligotyping or MIRU-VNTR analysis, some of which are more suitable for molecular epidemiology studies. However, since these methods are subject to a certain degree of homoplasy, other criteria have been chosen to assign lineages. These are based on targeting robust and specific SNPs for each lineage. Here, we propose two newly designed multiplex targeting methods—both of which are single-tube tests—to optimize the assignation of the six main lineages in MTB. The first method is based on ASO-PCR and offers an inexpensive and easy-to-implement assay for laboratories with limited resources. The other, which is based on SNaPshot, enables more refined standardized assignation of lineages for laboratories with better resources. Both methods performed well when assigning lineages from cultured isolates from a control panel, a test panel, and a problem panel from an unrelated population, Mexico, which included isolates in which standard genotyping was not able to classify lineages. Both tests were also able to assign lineages from stored isolates, without the need for subculture or purification of DNA, and even directly from clinical specimens with a medium-high bacilli burden. Our assays could broaden the contexts where information on lineages can be acquired, thus enabling us to quickly update data from retrospective collections and to merge data with those obtained at the time of diagnosis of a new TB case.

## Introduction

Genotyping and genomic analysis have enabled us to differentiate between *Mycobacterium tuberculosis* (MTB) strains with varying degrees of discrimination.

The magnitude of discrimination differs according to the objective of the analysis and should be greater when attempting to accurately define recent transmission clusters. In this context, population-based molecular epidemiology was initially based on IS6110 RFLP analysis [1], which was later substituted by MIRU-VNTR analysis [2]. The recent expansion of whole genome sequencing (WGS) means that recent transmission clusters are now being defined using genomic epidemiology, which is based on the quantification of the number of differential single-nucleotide polymorphisms (SNPs) between circulating strains [3].

However, this shift from population-based epidemiology to global epidemiology or phylogeographic analysis requires discriminatory power to be lower, since the objective of the approach is to identify major lineages families instead of clones/strains. In this context, other less discriminatory genotyping strategies, such as spoligotyping, have been extensively used [4] and various platforms and global databases compile extensive collections of isolates with the associated lineage or enable automatic assignation of lineages based on spoligotype or VNTR patterns [5–8].

Current genotyping approaches tend towards convergent evolution, which could lead to incorrect assignation of lineages [9,10]. On the other hand, using SNPs to classify lineages has proven to be the most robust approach, because SNPs show a low degree of homoplasy [10]. The classification of MTB complex into lineages based on either long sequence polymorphisms or SNPs has been modified over time [11]. However, the approaches used have led to the classification of MTB and *M*. *africanum* into six lineages, which have recently grown with the recent description of lineage 7 [12,13]. The distribution of lineages varies throughout the world: some, such as lineage 4, are widely distributed, whereas others are restricted to a specific geographic area (eg, Ethiopia for lineage 7). In addition, differences in virulence associated with diminished immune responses, more severe disease, and enhanced transmission have been described for certain lineages [12]. Therefore, optimal classification of lineages will help us to understand the mechanisms of host–pathogen interactions in TB.

The classification of lineages 1–6 can be supported using a set of six robust markers, which have proven to be lineage-specific in a wide sample of WGS data representative of the global diversity of MTB complex [14]. This set paves the way for the design of novel assays to optimize lineage assignation in MTB complex. Our objective was to design novel assays that could i) provide a simplified response (simultaneous interrogation for all SNPs in single-tube tests), ii) adapt to settings with different resources, and iii) be suitable not only for standard application on cultured isolates, but also for fast direct analysis on retrospective collections or on clinical specimens. We propose to bring phylogenetic analysis closer to the time of diagnosis and to enable classification of lineages in low-resource settings where MTB is not routinely cultured.

## Materials and methods

### Strain selection/sputum samples

The control panel included six strains, each of which was representative of lineages 1–6. The strains corresponded to clinical isolates whose lineage had been assigned by identification of the corresponding marker lineage SNPs [14] using Sanger sequencing.

The test panel included 40 clinical MTB isolates from a convenience sample, (selected from the period 2000–15) from three institutions located in different parts of Spain, where the populations are characterized by varying profiles of immigrant nationalities (20 isolates from Hospital General Universitario Gregorio Marañón Madrid [central Spain]; 14 isolates from Complejo Hospitalario Torrecárdenas, Almería [southeast Spain], and 11 isolates from Hospital Cliníc, Barcelona [northeast Spain]). We examined as wide a distribution of nationalities as possible to increase the variability of the lineages.

The problem panel included purified DNA from a collection of 400 isolates (January 2014-February 2016) from Veracruz, Mexico, which were obtained within a molecular epidemiology study (manuscript in preparation). Veracruz has a population of 7 million inhabitants, and around 10% of all cases of TB in Mexico are diagnosed in this state. We randomly selected 60 isolates from the collection. (27 in which lineage assignation based on spoligotyping [4] and MIRU-VNTR 24 –loci [15] was not possible and 33 isolates where the lineage was assigned successfully).

We also selected 19 stain-positive respiratory specimens for analysis. Our selection was taken from the remnants for the decontaminated sputa of the specimens received in the diagnostic laboratory that had been both stain- and culture-positive for MTB.

### Lineage assignation

Lineages were preassigned based on Spoligotype and/or MIRU-VNTR data following the instructions from TBminer platform, which provides the assignations according to SITVITWEB, MIRU-VNTRPlus, Borile_AffinityPropagation and TBlineage classifications, and offers a final consensus assignation [8].

In order to assign lineages based on lineage marker SNPs, we used the six SNPs reported by Stucki et al ([14], Table 1) as targets in our assays. These SNPs map in essential genes and are synonymous. Consequently, their stability was maximized, since they are highly unlikely to be involved in selective pressure processes.

**Table 1:** Lineage marker SNPs. [14].

Marker SNP	Position	Nucleotide change
SNP 1 (Lineage 1)	Rv3597682	G/A ^a^
SNP 2 (Lineage 2)	Rv3304966	C/T ^a^
SNP 3 (Lineage 3)	Rv4266647	A/G
SNP4 (Lineage 4)	Rv2154724	T/G ^a^
SNP 5 (Lineage 5)	Rv1377185	G/C
SNP 6 (Lineage 6)	Rv378404	G/A

^a^ The reference nucleotide corresponds to the one in the 3’-5’ strand

### DNA extraction

One milliliter of either cultured MTB isolates or decontaminated sputum (NaLC-NaOH 2% method [16] was extracted using a column-based purification method (QIAamp DNA minikit protocol; Qiagen, Courtabouef, France) and eluted in 60–100 μl (isolates) or 40–60 μl (sputa) of AE buffer.

### Allele-specific oligonucleotide multiplex PCR (ASO-PCR) assay

The final conditions for the ASO-PCR assay (multiplex format) were as follows: 1X PCR Gold buffer (Applied Biosystems [AB], Foster City, CA, USA); 1.8 mM MgCl_2_; 0.2 μM primers each for lineage 2, lineage 3, lineage 4, and lineage 6; and 0.3 μM primers each for lineage 1 and lineage 5 (Table 2); 200 μM dNTPs; 0.5 μl DMSO; and 0.4 μl AmpliTaq Gold (Applied Biosystems, Foster City, California, USA) in a final reaction volume of 50 μl. The PCR was run as follows: 95°C for 10 minutes followed by 29 cycles (95°C for 1 minute, 62°C for 1 minute, and 72°C for 1 minute, and a final extension step at 72°C for 10 minutes. The GeneAmp PCR system 9700 thermal cycler was used. Amplification products were sized using standard agarose electrophoresis.

**Table 2:** Multiplex ASO-PCR primers.

Primer	Targeted SNP	Size (bp)	Primer sequence (5´→ 3´)
Lineage 1	G/A	95	F: GAG GAT GTT CGC GCC GA R: TCC AGC AGC ACC ACG AC
Lineage 2	C/T	345	F: TCA ACC TGT ACC ACC GCA C R: CGG CGT ATG GGA AGT ACC C
Lineage 3	A/G	434	F: GTT GCA TTC CTA CGA GTT CAC C R: CGC CAC GAA CCC TGT CA
Lineage 4	T/G	520	F: CAG CCT TAA GAG CCA GAT CCT R: ACC TAC CAG CAC CGT CAT C
Lineage 5	G/C	183	F: ATC GTT GGC GTG GAC CTC R: GAA GAA CAC CCC GGC CAC
Lineage 6	G/A	267	F: ATA TCG GTT CGG CGG GC R: CGA CCG AAT GCT TGT ACT GC

Size: amplicon size. F: Forward; R: Reverse.

### Multiplex SNaPshot assay

The assay consisted of a multiplex PCR to obtain the six amplicons, including the region where each lineage marker SNP mapped (Table 3). Each amplicon was subsequently interrogated by a selective extension reaction from a primer whose 3′ end mapped only one nucleotide before the coordinate for the marker SNP (Tables 4 and 1). Since the marker SNPs are biallelic, one of each two alternative dideoxynucleotide terminators is incorporated in the extension reaction. Each alternative ddNTP is labelled with a different fluorophore, which makes it possible to indirectly identify the allele present in the target, depending on the fluorophore in the extension reaction.

**Table 3:** SNaPshot multiplex PCR primers.

Primer	Primer sequence (5’→ 3’)
Lineage 1	F: CAC ATC AGG TAA TCG GCA AAC R: CAG TCA GCA TAC GGC ATG G
Lineage 2	F: CTG GAG GTC AGT TGC GGA CAC R: GTC GAC CAG GTC CCG TGA CTT
Lineage 3	F: CAG CCG CTG AAG TCG TCC T R: CAG TCA GCA TAC GGC ATG G
Lineage 4	F: TGC TTT CTC TAT GGC GGC ACA R: CGA GGA ATT GGC CGA CGA GT
Lineage 5	F: CGT GCG GTC GAC TAT CTA TCC R: GTG ATC AGC CCG AAG AA
Lineage 6	F: TTT GCT GTG CGC TGC GGT ATG R: TTG TTG ACG CCC ATC ACG AC

F: Forward; R: Reverse.

**Table 4:** SNaPshot extension primers.

Lineage	Targeted SNP	Primer sequence (5’→ 3’)
Lineage 1	G/A	AAA AAA AAA AAA ATC AGA TCG ACA AGG GCG AC
Lineage 2	C/T	AAA AAA AAA AAA AAA AAA AAA AAA AAA AAA AAA AAA AAA AAA AAA AAA AAA AAA ATC GGC GTA TGG GAA GTA CC
Lineage 3	A/G	AAA AAA AAA AAA AAA AAA AAA AAG CGC CAC GAA CCC TGT C
Lineage 4	T/G	AAA AAA AAA AAA AAA AAA AAA AAA AAA AAA AAA AAA CAG CCT TAA GAG CCA GAT CC
Lineage 5	G/C	AAA AAA AAA AAA AAA AAA AAA AAA AAA AGT GAC CCG TTC AAC CTG CAT
Lineage 6	G/A	AAA AGC GAG AAC CTG CAA ATC CC

The multiplex PCR was performed in a final volume of 50 μl constituted by 1X PCR Gold buffer (Applied Biosystems [AB], Foster City, CA, USA), 2 mM MgCl_2_, 200 μM dNTPs, 0.4 μM for each primer, and 0.4 U of AmpliTaq Gold DNA polymerase (AB). Thermal cycling consisted of a denaturation step at 95°C for 10 minutes, followed by 27 cycles (95°C for 1 minute, 57°C for 1 minute, 72°C for 1 minute), and a final extension step at 72°C for 10 minutes.

The selective extension reaction was carried out in a final volume of 12 μl containing 5 μl of SNaPshot Multiplex Ready Reaction Mix (Applied Biosystems Co., USA), 5 μl of the multiplex PCR product (cleaned by USB ExoSAP-IT PCR Product Cleanup), and 0.4 μM of each minisequencing primer (Table 4). Selective extension was performed by 25 cycles of: denaturation at 96°C for 10 seconds, annealing at 57°C for 5 seconds, and extension at 60°C for 30 seconds. Unincorporated ddNTPs were removed by addition of 1 U of alkaline phosphatase (USB Shrimp Alkaline Phosphatase) to the mixtures and incubation at 37°C for 1 hour.

Ten microliters of the final product was sized and analyzed by capillary electrophoresis in a 3130xl Genetic Analyzer (polymer POP-7, 1200 standard, LIZ SS; Applied Biosystems, Foster City, CA, USA). Alleles were called using GeneMapper, version 4.0.

### Sanger sequencing

Lineages classified by applying the tests in the evaluation were finally confirmed by identification of the corresponding SNPs using Sanger sequencing. Confirmation was not possible with the analysis performed directly on sputa owing to material restrictions and, therefore we confirmed the lineage on the corresponding cultured isolates.

PCR (simplex format) was performed in a final volume of 50 μl: 5 μl DNA, 5U AmpliTaq Gold DNA polymerase LD (Applied Biosystems, Foster City, CA, USA), reaction buffer (B1OX) 1X, dNTPs 200 μM, 0.4 μM each primer, 1% mM DMSO, and 2 mM MgCl_2_. The thermal conditions were 95°C for 10 minutes and 27 cycles: 95°C for 1 minute; 57°C for 1 minute; and 72°C for 1 minute followed by 10 minutes at 72°C. The PCR product was sequenced using the Sanger protocol in a 3130xl Genetic Analyzer, (polymer POP-7, 1200 standard LIZ SS; Applied Biosystems, Foster City, CA, USA). Data were visualized and sequences interpreted using FinchTV Version 1.4.0 (Geospiza).

## Results

### Optimization and evaluation of the ASO-PCR and SNaPshot assays on control strains

The first step in the optimization process consisted of ensuring the following: i) correct amplification for the six regions targeted in the multiplex reactions; ii) appropriate size distribution for the amplicons/products; and iii) accurate discrimination of results between the six lineages interrogated. We went through these steps using a control panel including a clinical isolate representative of each of the six lineages analyzed (S1 Table).

After a performing a pre-evaluation analysis involving screening for the best Mg^2+^ and primer concentration and running a gradient of annealing temperatures, the optimal analysis conditions for the multiplex ASO-PCR and SNaPshot assays were set (see Methods).

**Fig 1:**
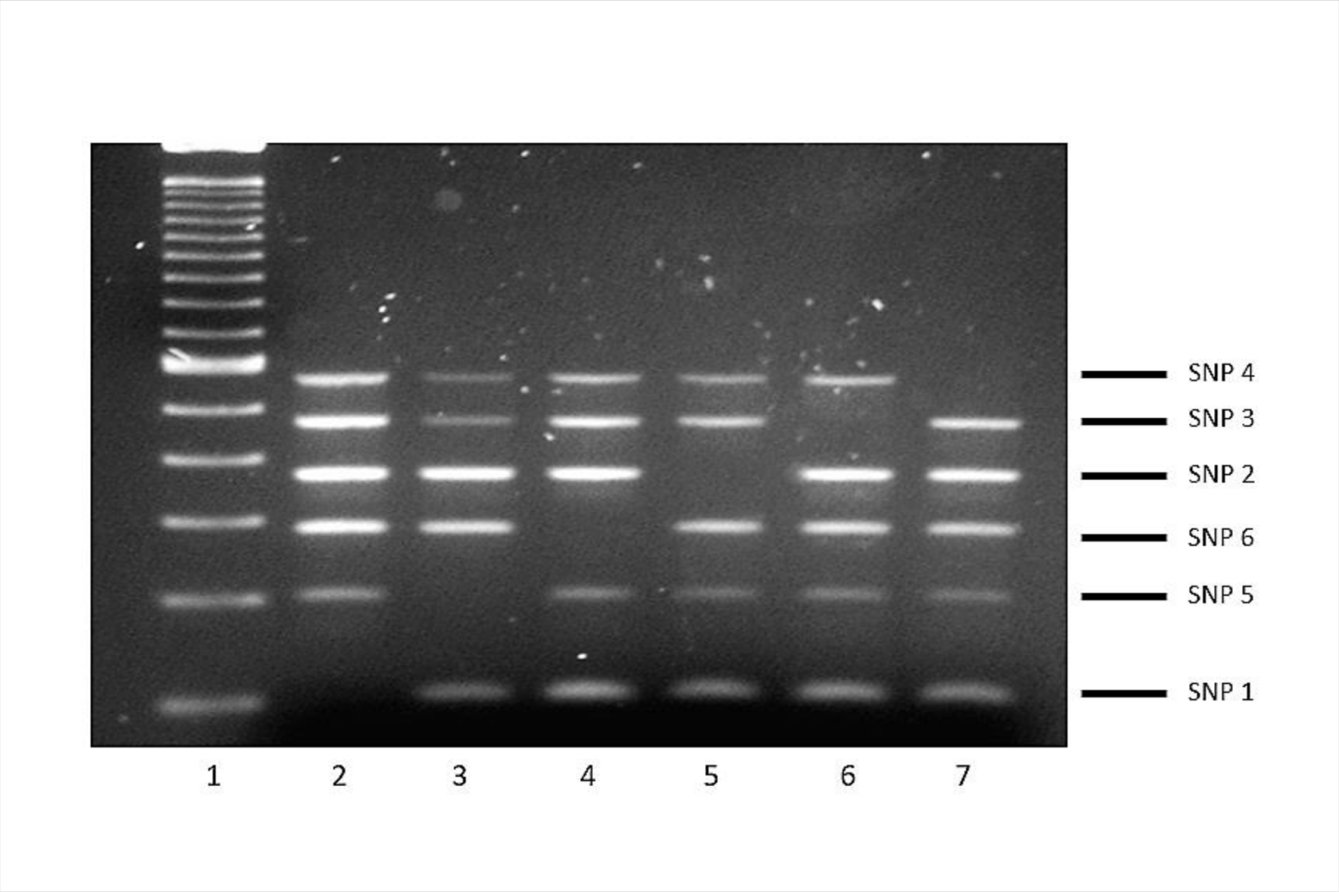
Patterns obtained in multiplex ASO-PCR with the control panel. Lane 1, 100-bp ladder; lanes 2–7, lineages 1, 5, 6, 2, 3, and 4. The amplicon corresponding to each lineage marker SNP and its size can be seen at the right side of the gel.

For ASO-PCR, the intensity and sizing of the amplification products enabled them to be detected and separated appropriately using standard electrophoresis. The selective performance of the test was optimal: whenever the corresponding lineage-marker SNP interfered with a single PCR (3′ ends of the selective primers were designed to coincide with the coordinate where the SNP mapped), the corresponding amplification was completely impaired, thus leading to five amplicon patterns, with a single amplicon missing from each of the lineages in the test panel (Fig 1).

**Fig 2:**
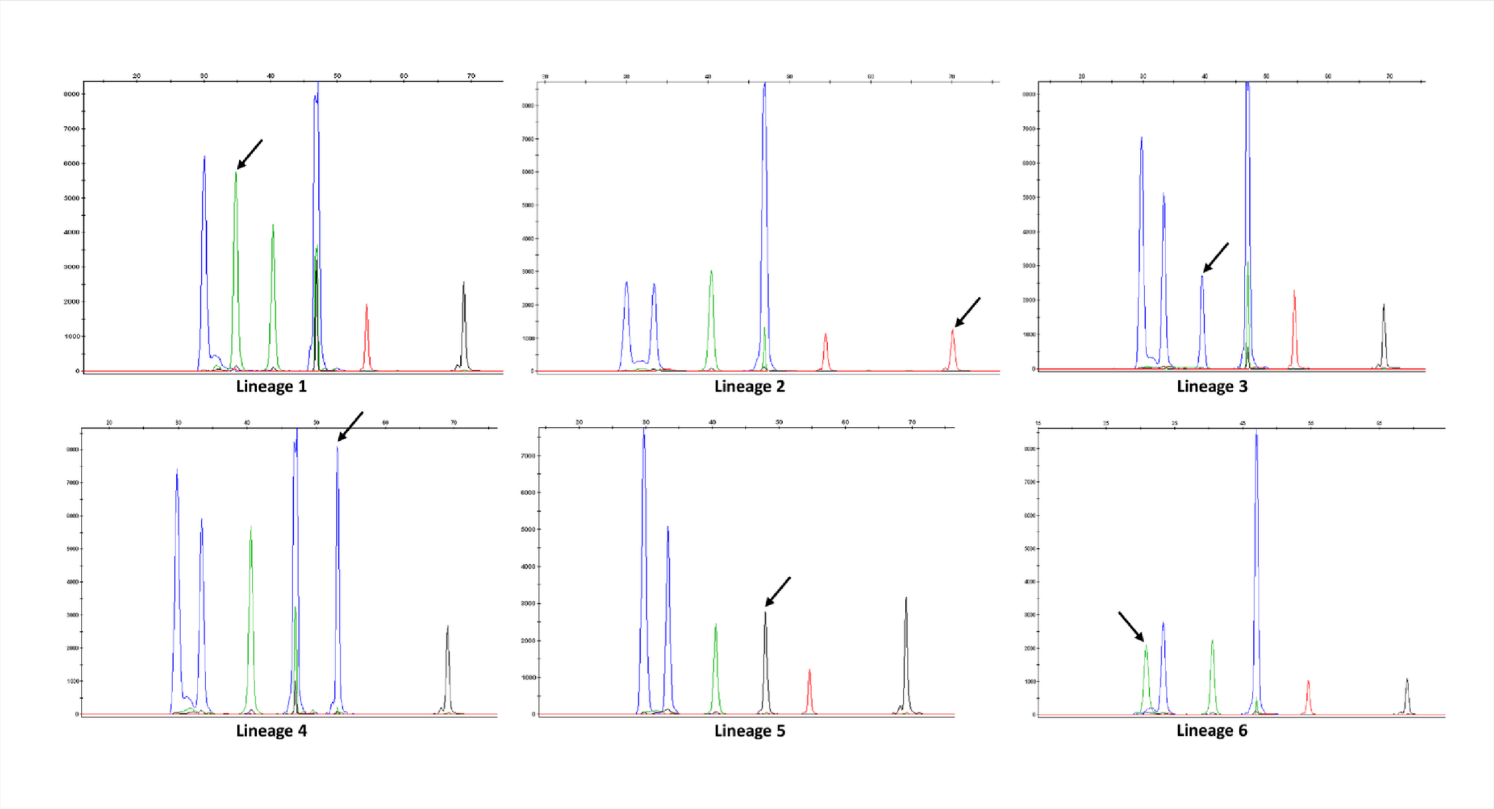
Patterns obtained in SNaPshot with the control panel. Each peak corresponds to an extended product in the following order from left to right (lineages 6, 1, 3, 5, 4 and 2). The arrow indicates the peak interrogated for each of the lineages. For each peak, two different labelled ddNTPs can be included alternatively in the extension reaction depending on the allele present in the amplicon. Depending on the absence/presence of the marker SNP for each corresponding peak, the two alternative colors expected for each lineage, are as follows: Lineage 1: blue/green; lineage 2: black/red; lineage 3: green/blue; lineage 4: red/blue; lineage 5: blue/black; and lineage 6: blue/green. Based on these features, the colour pattern (from left to right) expected for each lineage would be: Lineage 1: blue, green, green, blue, red, black, Lineage 2: blue, blue, green, blue, red, red, Lineage 3: blue, blue, blue, blue, red, black, Lineage 4: blue, blue, green, blue, blue, black, Lineage 5: blue, blue, green, black, red, black and Lineage 6: green, blue, green, blue, red, black.

In the case of SNaPshot, once optimized the multiplex PCR (see Methods) that rendered the six amplicons including each of the six SNPs to be interrogated, we designed progressively extended primers (using polyA tails, Table 4) for the selective extension reactions. The length of each primer indicated the lineage-specific SNP that we were interrogating (Table 4), whereas the fluorophore/color of the nucleotide included in each extension reaction allowed us to determine the lineage (Fig 2).

### Evaluation on a test panel of cultured isolates

The next step was to evaluate both assays on a test blind panel including purified DNA from a collection of 40 cultured isolates. In 37 cases, the lineage had been previously assigned from the spoligotype and/or MIRU-VNTR data (S1 Table) (two to lineage 1, six to lineage 2, two to lineage 3, seventeen to lineage 4, nine to lineage 5, one to lineage 6, and three with no consensus). ASO-PCR enabled the correct assignation of lineages of all 37 isolates preasigned based on standard genotyping and SNaPshot in 36 (S1 Table). Both assays were able to assign lineage in the 3 isolates without a previous assignation. In all cases, the assignations were confirmed by identification of the lineage marker SNPs using DNA sequencing.

### Performance of the assays on a problem panel

Once the two assays had been evaluated on test panels, we applied them to another sample from an unrelated population. We analyzed a collection of 60 isolates obtained in Mexico (S1 Table), in which a first attempt to assign lineages based on the combined analysis of their spoligotypes and 24-loci MIRU patterns had failed in 27 cases (45%). The remaining 33 isolates had been correctly assigned. The lineage was correctly assigned by both assays in 29 of the 33 isolates with a preassigned lineage (27 isolates to lineage 4, one to lineage 1 and one to lineage 2). In the remaining four isolates (preassigned as lineage 4), the presence of inaccurate patterns precluded assignation by one or both assays (one of them was assigned only by SNaPshot; S1 Table). 23 of the 27 isolates without a pre-assigned lineage were now successfully assigned by either ASO-PCR and SNaPshot (all isolates to lineage 4 but one to lineage 2). Four isolates remained unassigned after application of both tests owing to unspecific bands in the ASO-PCR pattern and lack of extension products in SNaPshot.

### Evaluation using crude extracts

**Fig 3:**
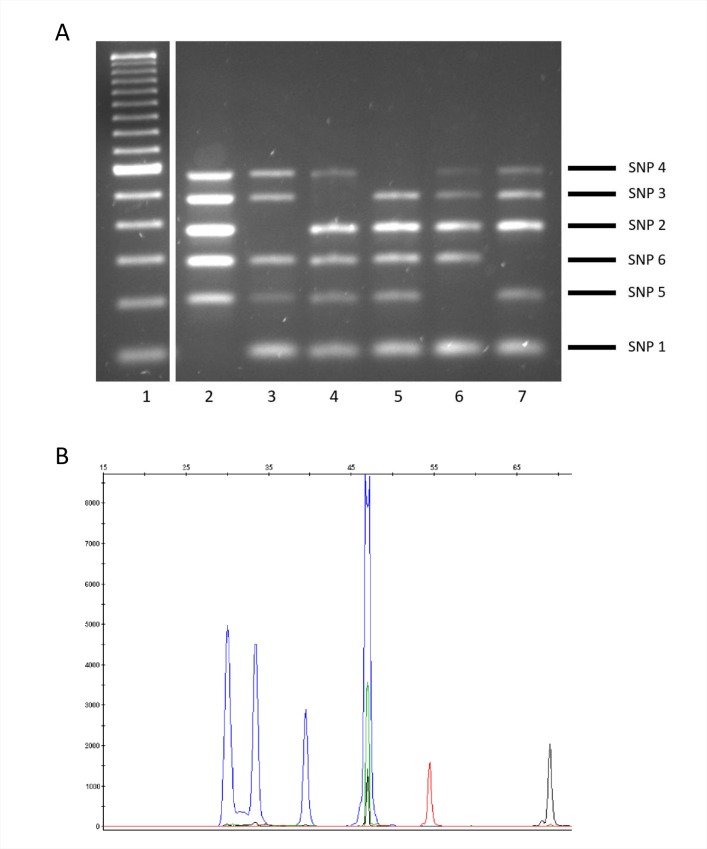
Patterns obtained when multiplex ASO-PCR (panel A) or SNaPshot (panel B) was applied on crude extracts from stored isolates. (A) lane 1: 100-bp ladder, lanes 2–7: lineages 1, 2, 3, 4, 5, and 6. The amplicon corresponding to each lineage marker SNP and its size are shown on the right side of the gel. (B) Representative result for lineage 3.

To evaluate whether the tests could be applied directly on stored MTB isolates for fast analysis of retrospective collections, we checked whether lineages could be assigned directly on the supernatant from boiled frozen stored isolates, that is, without subculture or DNA purification. Assignation was correct for a representative of each lineage when the test was applied directly (Fig 3A and 3B).

### Direct analysis on respiratory specimens

The possibility of assigning a lineage at diagnosis of a new case, without waiting until culture is available, was evaluated by directly applying the assays on a selection of 19 stain-positive respiratory specimens.

**Fig 4:**
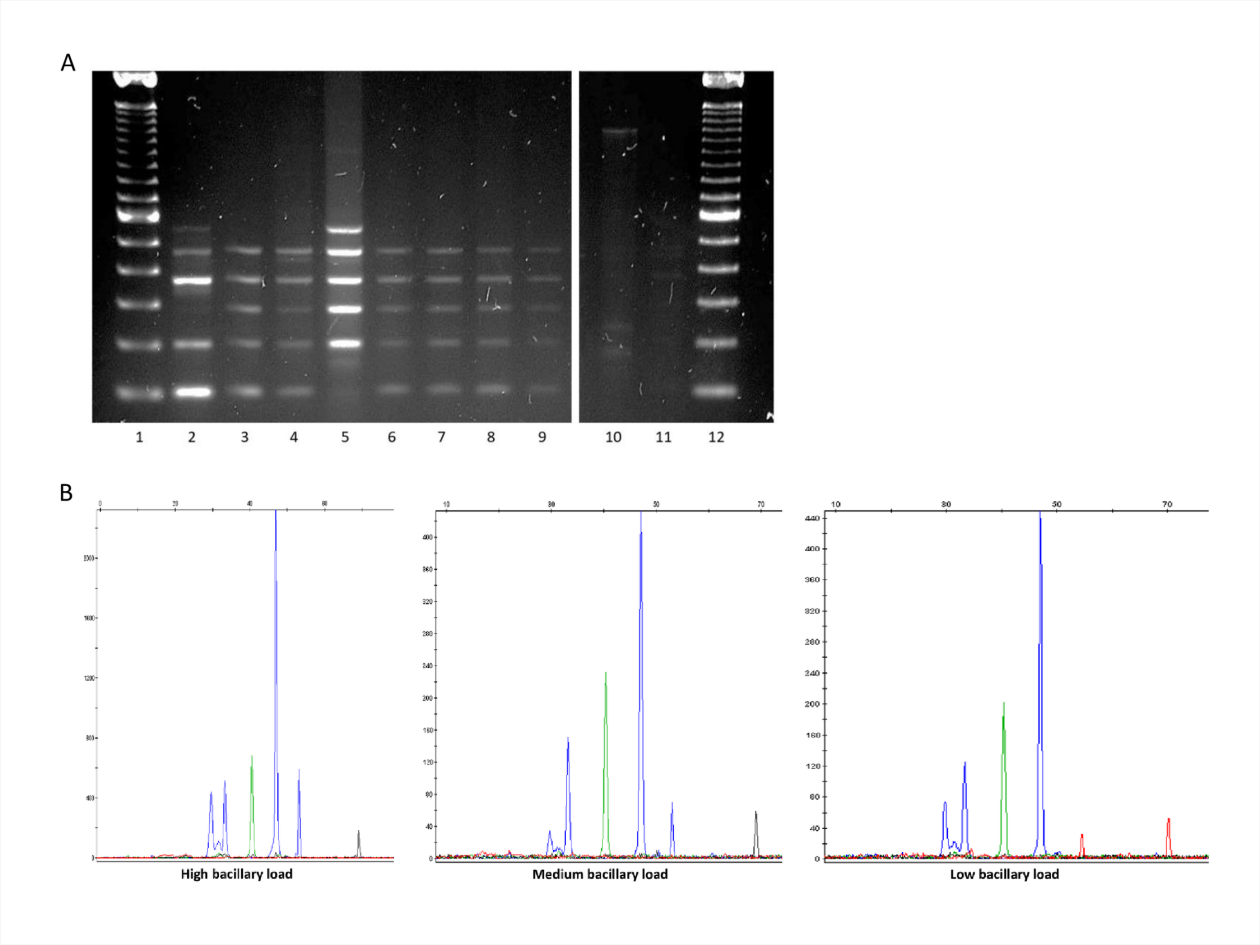
Patterns obtained when multiplex ASO-PCR (panel A) or SNaPshot (panel B) was applied directly on respiratory specimens. (A) lanes 1 and 12: 100-bp ladder; lane 2: DNA control for lineage 6; lanes 3–7: 3+ sputa (all lineages 4 except lineage 1 in lane 5); lanes 8–9: 2+ sputa (both lineage 4); lanes 10–11: 1+ sputa (uninterpretable results). (B) Representative panels for 3+ (lineage 4), 2+ (lineage 4), and 1+ (lineage 2) sputa.

ASO-PCR enabled us to assign the lineage from 12 out of 19 sputa samples (Fig 4A) (one to lineage 1 and 11 to lineage 4), most of which had a high and medium bacterial load (3+ and 2+) and one a low bacterial load (1+). SNaPshot assigned the lineage in 14 sputa samples (Fig 4B) (those assigned by ASO-PCR and, in addition, one lineage 2 in a 3+ specimen and another lineage 4 in a 1+ specimen). The isolates cultured from 9 of the 14 sputa with an assignation were available and the direct assignation on sputa was consistent with the lineage assignation based on MIRU-VNTR data from the corresponding isolates.

### Detecting mixed infections

**Fig 5:**
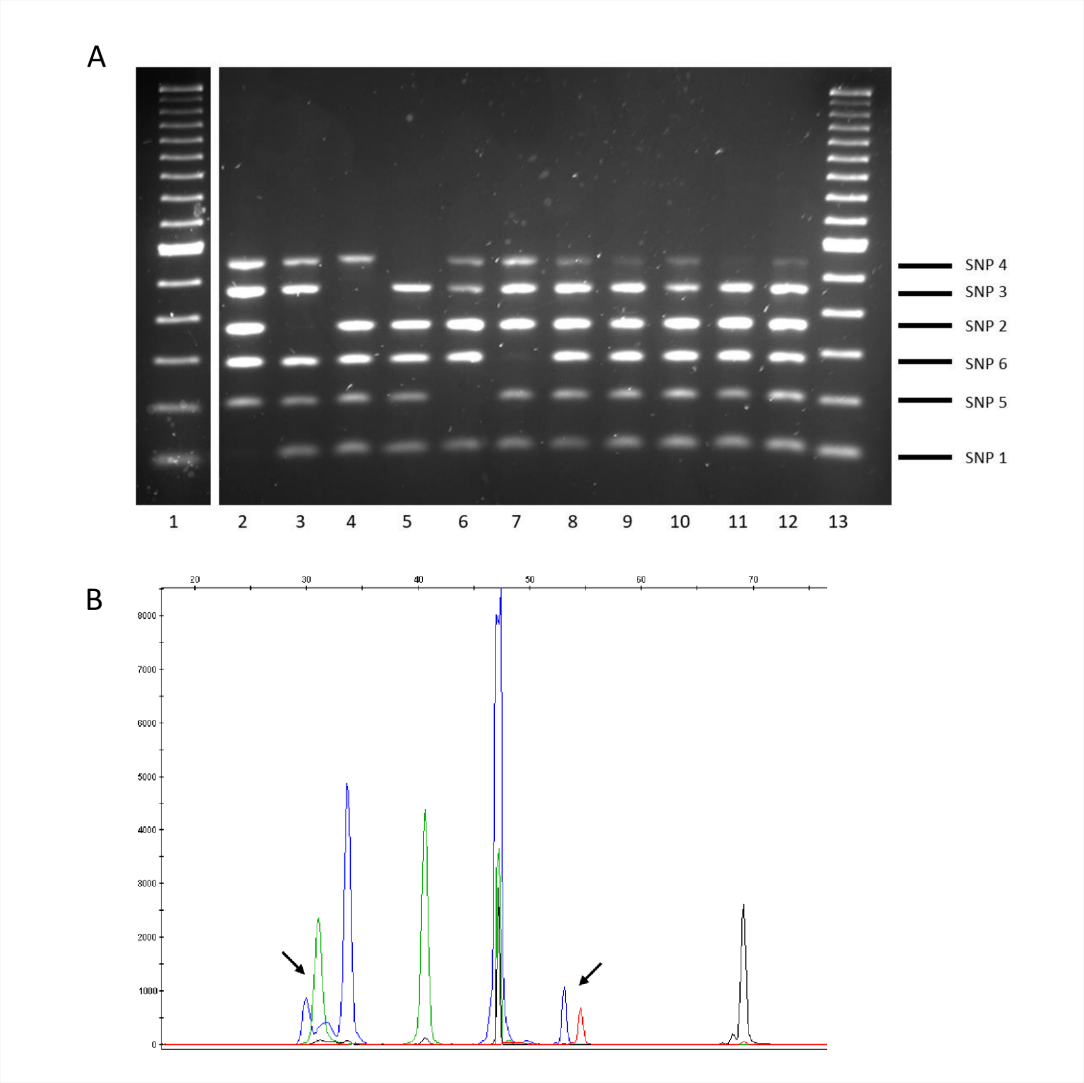
Patterns obtained when multiplex ASO-PCR (panel A) or SNaPshot (panel B) was applied to samples spiked with two different lineages. (A) Lanes 1 and 13: 100-bp ladder; lanes 2–7: controls for lineages 1–6; lanes 8–12: 50:50 mixtures comprising lineage 4 and lineages 1, 2, 3, 5, and 6. (B) Representative panel for a mixture comprising lineages 6 and 4. The arrows indicate the presence of double peaks (each one with a different color) corresponding to the extension of the two alternative alleles.

The last feature evaluated was whether the assays could identify mixed infections involving two different lineages. We applied the assays to laboratory mixes (50:50) comprising a lineage 4 isolate (the most prevalent in our context) and each of the other five remaining lineages under analysis. In contrast to the five-amplicon pattern expected when assaying a single lineage, ASO-PCR showed all six amplicons because of the overlap between the patterns expected for the two lineages involved (Fig 5A). Therefore, the observation of this singular six-band amplification pattern alerted us to the presence of a mixed infection with more than one lineage. However, given the design of our ASO-PCR assay, we were unable to determine the two specific lineages involved in the mixed infections. On the contrary, the rationale underlying the SNaPshot design enabled us to resolve the limitations of ASO-PCR. The same 50:50 mixes were then analyzed by SNaPshot, which succeeded in identifying not only mixed infections, but also the specific lineages according to the color patterns of the fluorophores included in the extension reactions (Fig 5B).

## Discussion

The degree of discrimination to be applied when identifying a microorganism depends on the aim of the study. In MTB, the microorganism is characterized to species level in the diagnostic setting, even though this is the lowest degree of discrimination. At the other extreme, molecular epidemiology applied to track transmission clusters at the population level demands maximum discriminatory power.

Between these degrees of characterization, which enable diagnosis to be confirmed or transmission to be assessed, we find intermediate levels of discrimination that can be applied in specific circumstances. Such is the case of lineage assignation, which is useful for phylogenetic analysis and surveying the global distribution of MTB at the macro-population level. The consequences of genome diversity in MTB for virulence have been reported [17], with some lineages, such as lineage 2 and lineage 4, being more virulent than other, more geographically restricted lineages [12]. Therefore, the development of methods to optimize lineage classification in MTB would improve the analysis of host–pathogen interactions.

The most robust approach for assigning lineages is that based on identification of lineage marker SNPs, which can be identified directly using methods such as standard direct Sanger sequencing and even more advanced approaches such as WGS. However, adapting laboratory tests to indirectly assess the presence of this set of marker SNPs is more convenient. The various tests and algorithms that have been developed to optimize lineage classification target some of the SNPs previously used for phylogenetic purposes, other genetic elements [18], or a newly proposed set of SNPs [19]. To our knowledge, only one attempt has been made to develop tests targeting the current set of six robust SNPs [14]. The authors, who originally proposed the set of markers, designed a refined single-tube analysis. However, this approach was based on a Luminex device, which is still uncommon in the microbiology laboratory. The authors also designed a second test based on PCR that is well adapted to more widely available platforms (real-time PCR) it was based on singleplex assays, thus creating the need for six independent reactions to classify the lineage.

Current limitations to finding a wider variety of tests to classify lineages led us to attempt a new approach. We defined three strict requirements for developing two new tests. First, both had to have a single-tube reaction design to ensure simplicity and reduce costs. Second, each had to be adapted to fit laboratories with different resources. The multiplex ASO-PCR–based test is inexpensive and easy to implement, with no need for complex equipment, and is especially suitable for laboratories with basic resources. The SNaPshot analysis–based approach offers more accurate and semiautomatic assignation, albeit at a higher cost and requiring capillary electrophoresis. Finally, the new tests had to be able to respond to demands that were broader than the standard classification of cultured isolates, that is, for fast direct analysis of retrospective isolate collections without the need for subculture or direct application on clinical respiratory specimens.

The performance of the two assays was equivalent with cultured isolates, and both were able to correctly assign 100% of cases, thus ensuring their usefulness when applied systematically to prospectively characterize the lineages circulating in a population. In fact, we evaluated the tests on a challenging sample from Mexico in which a high percentage of cases could not be assigned by combining spoligotyping and MIRU-VNTR typing. The tests succeeded in assigning a lineage in most of those that were previously unassigned. The alternative standard approach to assign lineages based on MIRU-VNTR data would have required greater experimental efforts (i.e. 24 PCRs to obtain the MIRU-VNTR pattern) to have the assignation for all the patterns. We also proved that the assays could quickly assign lineages by direct analysis on the stored isolates, without the need for subculture or purifying DNA. We believe that the assays would provide a rapid and inexpensive means of obtaining data on the distribution of lineages in settings where this information is not updated.

Genotyping for diagnosis of MTB infection is undergoing a transformation. Culture to identify the species and subsequent subculture to determine the susceptibility pattern are being replaced by commercial molecular tests that make it possible to identify MTB and assess the presence of the most common resistance mutations directly on clinical specimens. In short, detailed characterization of the strain is possible when TB is diagnosed, that is, when acid-fast bacilli are observed in microscopy.

Following the same approach to in-depth characterization of the strain at diagnosis, we evaluated the possibility of assigning the lineage directly on respiratory specimens. Such an approach would prove useful when an unknown lineage has been identified as recently imported in a population and it is crucial to fast-track the emergence of secondary cases caused by this lineage. Direct assignation is frequently requested when a high-risk strain, such as those belonging to the Beijing sublineage (within the, ***East-Asian*, *lineage 2***), which is frequently involved in severe outbreaks [20,21], is imported into a population. The two assays allowed us to assign lineages directly on clinical specimens, although they were restricted to specimens with medium-high bacterial content.

As a collateral analysis, we aim to evaluate the usefulness of our tests when mixed infections are suspected. Given the feature targeted—lineages—we are limited by the fact that the tests will be only be able to detect mixed infections when different lineages are involved. Both tests proved able to efficiently identify mixed infections from mixtures artificially generated in the laboratory. Given the specific design of our ASO-PCR, only SNaPshot would also be able to determine the two specific lineages involved in each mixed infection. This ability could be considered useful only in very unusual circumstances. However, in settings with a high disease burden, both mixed infections and those involving different lineages are common [22,23]. Our assays would add value in these settings.

In summary, we developed two tests to optimize the assignation of lineages in MTB. Each test is adapted to a different laboratory resource profile, and both tests increase the possibility of updating the composition of lineages in a population and performing fast prospective assignation, even directly on clinical specimens, thus expanding the information we can currently obtain when a new TB case is diagnosed.

## Supporting information

S1 Table (PDF)

## References

[1] van Embden JD, Cave MD, Crawford JT, Dale JW, Eisenach KD, et al (1993) Strain identification of Mycobacterium tuberculosis by DNA fingerprinting: recommendations for a standardized methodology. J Clin Microbiol 31: 406–409. 8381814 10.1128/jcm.31.2.406-409.1993 PMC262774

[2] de Beer JL, Akkerman OW, Schurch AC, Mulder A, van der Werf TS, et al (2014) Optimization of standard in-house 24-locus variable-number tandem-repeat typing for Mycobacterium tuberculosis and its direct application to clinical material. J Clin Microbiol 52: 1338–1342. doi: 10.1128/JCM.03436-13 24501023 10.1128/JCM.03436-13 PMC3993658

[3] Walker TM, Lalor MK, Broda A, Saldana Ortega L, Morgan M, et al (2014) Assessment of Mycobacterium tuberculosis transmission in Oxfordshire, UK, 2007–12, with whole pathogen genome sequences: an observational study. Lancet Respir Med 2: 285–292. doi: 10.1016/S2213-2600(14)70027-X 24717625 10.1016/S2213-2600(14)70027-X PMC4571080

[4] Kamerbeek J, Schouls L, Kolk A, van Agterveld M, van Soolingen D, et al (1997) Simultaneous detection and strain differentiation of Mycobacterium tuberculosis for diagnosis and epidemiology. J Clin Microbiol 35: 907–914. 9157152 10.1128/jcm.35.4.907-914.1997 PMC229700

[5] Weniger T, Krawczyk J, Supply P, Niemann S, Harmsen D (2010) MIRU-VNTRplus: a web tool for polyphasic genotyping of Mycobacterium tuberculosis complex bacteria. Nucleic Acids Res 38: W326–331. doi: 10.1093/nar/gkq351 20457747 10.1093/nar/gkq351 PMC2896200

[6] Allix-Beguec C, Harmsen D, Weniger T, Supply P, Niemann S (2008) Evaluation and strategy for use of MIRU-VNTRplus, a multifunctional database for online analysis of genotyping data and phylogenetic identification of Mycobacterium tuberculosis complex isolates. J Clin Microbiol 46: 2692–2699. doi: 10.1128/JCM.00540-08 18550737 10.1128/JCM.00540-08 PMC2519508

[7] Demay C, Liens B, Burguiere T, Hill V, Couvin D, et al (2012) SITVITWEB—a publicly available international multimarker database for studying Mycobacterium tuberculosis genetic diversity and molecular epidemiology. Infect Genet Evol 12: 755–766. doi: 10.1016/j.meegid.2012.02.004 22365971 10.1016/j.meegid.2012.02.004

[8] Aze J, Sola C, Zhang J, Lafosse-Marin F, Yasmin M, et al (2015) Genomics and Machine Learning for Taxonomy Consensus: The Mycobacterium tuberculosis Complex Paradigm. PLoS One 10: e0130912 doi: 10.1371/journal.pone.0130912 26154264 10.1371/journal.pone.0130912 PMC4496040

[9] Bouakaze C, Keyser C, de Martino SJ, Sougakoff W, Veziris N, et al (2010) Identification and genotyping of Mycobacterium tuberculosis complex species by use of a SNaPshot Minisequencing-based assay. J Clin Microbiol 48: 1758–1766. doi: 10.1128/JCM.02255-09 20220173 10.1128/JCM.02255-09 PMC2863856

[10] Comas I, Homolka S, Niemann S, Gagneux S (2009) Genotyping of genetically monomorphic bacteria: DNA sequencing in Mycobacterium tuberculosis highlights the limitations of current methodologies. PLoS One 4: e7815 doi: 10.1371/journal.pone.0007815 19915672 10.1371/journal.pone.0007815 PMC2772813

[11] Gagneux S, Small PM (2007) Global phylogeography of Mycobacterium tuberculosis and implications for tuberculosis product development. Lancet Infect Dis 7: 328–337. doi: 10.1016/S1473-3099(07)70108-1 17448936 10.1016/S1473-3099(07)70108-1

[12] Coscolla M, Gagneux S (2014) Consequences of genomic diversity in Mycobacterium tuberculosis. Semin Immunol 26: 431–444. doi: 10.1016/j.smim.2014.09.012 25453224 10.1016/j.smim.2014.09.012 PMC4314449

[13] Firdessa R, Berg S, Hailu E, Schelling E, Gumi B, et al (2013) Mycobacterial lineages causing pulmonary and extrapulmonary tuberculosis, Ethiopia. Emerg Infect Dis 19: 460–463. doi: 10.3201/eid1903.120256 23622814 10.3201/eid1903.120256 PMC3647644

[14] Stucki D, Malla B, Hostettler S, Huna T, Feldmann J, et al (2012) Two new rapid SNP-typing methods for classifying Mycobacterium tuberculosis complex into the main phylogenetic lineages. PLoS One 7: e41253 doi: 10.1371/journal.pone.0041253 22911768 10.1371/journal.pone.0041253 PMC3401130

[15] Supply P, Allix C, Lesjean S, Cardoso-Oelemann M, Rusch-Gerdes S, et al (2006) Proposal for standardization of optimized mycobacterial interspersed repetitive unit-variable-number tandem repeat typing of Mycobacterium tuberculosis. J Clin Microbiol 44: 4498–4510. doi: 10.1128/JCM.01392-06 17005759 10.1128/JCM.01392-06 PMC1698431

[16] Kubica GP, Dye WE, Cohn ML, Middlebrook G (1963) Sputum digestion and decontamination with N-acetyl-L-cysteine-sodium hydroxide for culture of mycobacteria. Am Rev Respir Dis 87: 775–779. doi: 10.1164/arrd.1963.87.5.775 13927224 10.1164/arrd.1963.87.5.775

[17] Niemann S, Supply P (2014) Diversity and evolution of Mycobacterium tuberculosis: moving to whole-genome-based approaches. Cold Spring Harb Perspect Med 4: a021188 doi: 10.1101/cshperspect.a021188 25190252 10.1101/cshperspect.a021188 PMC4292095

[18] Bergval I, Sengstake S, Brankova N, Levterova V, Abadia E, et al (2012) Combined species identification, genotyping, and drug resistance detection of Mycobacterium tuberculosis cultures by MLPA on a bead-based array. PLoS One 7: e43240 doi: 10.1371/journal.pone.0043240 22916230 10.1371/journal.pone.0043240 PMC3423362

[19] Homolka S, Projahn M, Feuerriegel S, Ubben T, Diel R, et al (2012) High resolution discrimination of clinical Mycobacterium tuberculosis complex strains based on single nucleotide polymorphisms. PLoS One 7: e39855 doi: 10.1371/journal.pone.0039855 22768315 10.1371/journal.pone.0039855 PMC3388094

[20] Johnson R, Warren R, Strauss OJ, Jordaan AM, Falmer AA, et al (2006) An outbreak of drug-resistant tuberculosis caused by a Beijing strain in the western Cape, South Africa. Int J Tuberc Lung Dis 10: 1412–1414. 17167961

[21] Caminero JA, Pena MJ, Campos-Herrero MI, Rodriguez JC, Garcia I, et al (2001) Epidemiological evidence of the spread of a Mycobacterium tuberculosis strain of the Beijing genotype on Gran Canaria Island. Am J Respir Crit Care Med 164: 1165–1170. doi: 10.1164/ajrccm.164.7.2101031 11673204 10.1164/ajrccm.164.7.2101031

[22] Dickman KR, Nabyonga L, Kateete DP, Katabazi FA, Asiimwe BB, et al (2010) Detection of multiple strains of Mycobacterium tuberculosis using MIRU-VNTR in patients with pulmonary tuberculosis in Kampala, Uganda. BMC Infect Dis 10: 349 doi: 10.1186/1471-2334-10-349 21143966 10.1186/1471-2334-10-349 PMC3004912

[23] Shamputa IC, Jugheli L, Sadradze N, Willery E, Portaels F, et al (2006) Mixed infection and clonal representativeness of a single sputum sample in tuberculosis patients from a penitentiary hospital in Georgia. Respir Res 7: 99 doi: 10.1186/1465-9921-7-99 16846493 10.1186/1465-9921-7-99 PMC1538999

