# Supplementary material for: Optimizing and accelerating the assignation of lineages in Mycobacterium tuberculosis using novel alternative single-tube assays
Source: PLoS One. 2017 Nov 1;12(11):e0186956. doi: 10.1371/journal.pone.0186956 (PMC5665510; doi:10.1371/journal.pone.0186956)
Supplement: S1 Table — (PDF) [file pone.0186956.s001.pdf]

Supplementary table

Panel	Sample	MIRUtype[a]	Spoligotype	Prediction[b]	ASO-PCR result	SNaPshot result
Control panel	5098279	5224341442218(10)4262223363	1101111111100001111001111111000000111111111	No consensus	L1	L1
Control panel	06126613bjn	263335444432647253213423	0000000000000000000000000000000000111111111	L2	L2	L2
Control panel	AL-4900506	27364542-234-48	1110000111111111111111100000000000101110111	L3	L3	L3
Control panel	AL-3012605	255432322122236132213423	1111111111111111111100001101111100001111111	L4	L4	L4
Control panel	BCN-31S	241442144233_22242224323		L5	L5	L5
Control panel	BCN-4S	242535356443546242223323		L6	L6	L6
Test panel	AL-1035506	234433342212423	1111111100011110111000000000000000001111111	L4	L4	L4
Test panel	AL-1828405bjn	27333544443265(10)	0000000000000000000000000000000000111111111	L2	L2	L2
Test panel	al-2038312	254323243232425252213423		L4	L4	L4
Test panel	AL-2079005	233433342212325	1111111100011111111000000000000000000011111	L4	L4	L4
Test panel	AL-2521305	253633233423626	1111111111111111111111111111110100001111111	L4	L4	L4
Test panel	al-2904512	254233343232623252113423		L4	L4	L4
Test panel	AL-3112703		1111111111111111111111000000000000000000000	L4	L4	L4
Test panel	al-3165012	255332322122236132213423		L4	L4	L4
Test panel	AL-3229903	234433442212223	1111111100011100111000000000000000000011111	L4	L4	L4
Test panel	al-3283512	454532233443437252113223		L4	L4	L4
Test panel	AL-3511807	123433233443537	1111111111111111111111111000000000000000000	No consensus	L4	L4
Test panel	AL-3735803	352332044232319	1110111100111111111111111111111100001111111	L4	L4	L4
Test panel	AL-3858305	253445324244237	1110000111111111110000000000000000111111111	L3	L3	L3
Test panel	AL-4011005bjn	27333544443265(10)	0000000000000000000000000000000000111111111	L2	L2	L2
Test panel	AL-4053203	234433342212425	1111100000000000011000000000000000000011111	No consensus	L4	L4
Test panel	AL-4087803	233433342212325	1111111100011111111000000000000000000011111	L4	L4	L4
Test panel	BCN-12R	231442144433_22242224333		L5	L5	L5
Test panel	BCN-13R	241442144413_22242224323		L5	L5	L5
Test panel	BCN-17R	241442144433_22242224323		L5	L5	L5

Test panel	BCN-32S	241442144433_22242223323		L5	L5	L5
Test panel	BCN-35S	231442443433_23242224323		L5	L5	L5
Test panel	BCN-36S	241442144433_22242223323		L5	L5	L5
Test panel	BCN-45S	241442144433_22242224323		L5	L5	L5
Test panel	BCN-66S	241462134433_22242224333		L5	L5	L5
Test panel	BCN-7S	241442144433_22242224323		L5	L5	L5
Test panel	BCN-82S	2425453564335662422233343		L6	L6	L6
Test panel	MFJ-06020070	253432232433435	1101111111111111111111111111111110100001111111	L4	L4	No assignable
Test panel	MGM-07177945		1101111111111111111101111111000010111111111	L1	L1	L1
Test panel	MPH-0200400000275	252533233432426	1111111111111111111111111111111110100001111111	L4	L4	L4
Test panel	MPH-06043974	255332342122226	1111111110110100011100001111011100001100111	L4	L4	L4
Test panel	MPH-06044028bjn	273335444432458	0000000000000000000000000000000000111111111	L2	L2	L2
Test panel	MPR-05163933		1110000111111111111100000000000000011111111	L3	L3	L3
Test panel	MPZ-04095333		1101111101111111111001111111000000011111111	No consensus	L1	L1
Test panel	MPZ-06046551	253533233331236	1111111111111111111111111110000001000011110111	L4	L4	L4
Test panel	MPZ-06080853		1111111111110111111111111111111110100001101111	L4	L4	L4
Test panel	MRC-04015937	523435248261664	1110000001111111111111001111000010111111111	L1	L1	L1
Test panel	MRC-06081357		1111111111111111111111111111111110100001100111	L4	L4	L4
Test panel	MSC-0552479 bjn	373335443434248	0000000000000000000000000000000000110111111	L2	L2	L2
Test panel	MSC-0587992bjn	27333544443265(10)	0000000000000000000000000000000000111111111	L2	L2	L2
Test panel	MSC-06063081bjn	274335444434672	0000000000000000000000000000000000111111111	L2	L2	L2
Clinical Specimen	11029844	253533233443534252211423		L4	L4	L4
Clinical Specimen	11123591	253233243232324262213423		L4	No assignable	L4
Clinical Specimen	14247455	254313243252325252213423		L4	L4	L4

Clinical Specimen	14255935	252422342122237162213423		L4	No assignable	No assignable
Clinical Specimen	15097617	252533233442427232113223		L4	No assignable	No assignable
Clinical Specimen	15121197	251343232232125252213423		L4	L4	L4
Clinical Specimen	15159127	5245342461(10)1865253223353		L1	L1	L1
Clinical Specimen	15204501	253333242232425252213223		L4	L4	L4
Clinical Specimen	15210819	252343242232325252213223		L4	L4	L4
Clinical Specimen	16054158	253533233432436252213423		L4	L4	L4
Clinical Specimen	16174439	251423542122336262113415		L4	L4	L4
Clinical Specimen	230863	Culture not available			L4	L4
Clinical Specimen	16174979	Culture not available			No assignable	No assignable
Clinical Specimen	228094	Culture not available			No assignable	No assignable
Clinical Specimen	14127170	Culture not available			L4	L4
Clinical Specimen	13206282	Culture not available			No assignable	L2
Clinical Specimen	12018069	Culture not available			L4	L4
Clinical Specimen	13003540	Culture not available			L4	L4
Clinical Specimen	13154732	Culture not available			No assignable	No assignable
Problem panel	402	263633242434624332314134	0000000000000000100000001000000100001111111	L4	L4	L4
Problem panel	403	263744343414426353225623	0111111111111111111111111111110100001111111	No consensus	L4	L4
Problem panel	405	2623331422324(12)5262213433	0000000000000000100000001000000100001111111	No consensus	L4	L4
Problem panel	410	253634353535337353314523	1111111111111111111111111111110100001111111	L4	L4	L4
Problem panel	413	451323442122437263213418	1111111111111111111100001111111100001111111	L4	L4	L4
Problem panel	415	251344343153424252214424	1111111111111111111111111111111100000000111	L4	L4	L4
Problem panel	416	255544633234339251213434	1111111111111111111111110000000000000000000	No consensus	L4	L4
Problem panel	417	251344343151424252212424	1111111111111111111111111111111100000000111	L4	L4	L4
Problem panel	418	443434532123337352223323	1101111000000001111110000111111100001111111	L4	L4	L4
Problem panel	420	252542433424549242224533	1111111111111111111111111111111100001111111	L4	L4	L4
Problem panel	421	265553644345449474436635	1111111111111111111011111111111100001111111	L4	L4	L4
Problem panel	423	271543433424448251214442	1111111111100110111111111111111100001110110	No consensus	L4	L4
Problem panel	431	233323442213337263212421	0111111100011111111100001111111100001111111	L4	L4	L4

Problem panel	432	253734333513527353314523	1111111111111111111111111111110100001111111	L4	L4	L4
Problem panel	440	264644344545328353314423	1111110011111111111111111111110100001111111	No consensus	L4	L4
Problem panel	451	243654742314434374325521	0000000000000100100000011111101000011111111	No consensus	L4	L4
Problem panel	454	254434633544328254224533	1110000000000111110111001111111100001111111	No consensus	L4	L4
Problem panel	460	271543433424443291252422	1111111111100110111111111111111100001110111	L4	L4	L4
Problem panel	463	263434233333738232213223	1111111111100111111111110000101000011101111	L4	L4	L4
Problem panel	466	263623232445637252213523	1111111111111111111111111000000100001111111	L4	L4	L4
Problem panel	471	233444342234335253223424	0011111111011111101111111111111100001111111	No consensus	L4	L4
Problem panel	482	263642133424524233214231	1111111110011111101111111111111100001111111	L4	L4	L4
Problem panel	484	263453242432624360314134	00000000000000000000000001000000100001111111	L4	L4	L4
Problem panel	485	253533233433435252313223	0000000000000000000000001111110100001111111	L4	L4	L4
Problem panel	490	25445163324432(10)254224433	1110000000000111110111001111111100001111111	No consensus	L4	L4
Problem panel	492	254434414440532361333524	1110000011111111111111111111110100001111111	No consensus	L4	L4
Problem panel	494	265844343535626273114423	1111111100011111111100001111111100001111111	No consensus	L4	L4
Problem panel	496	222533243403497252223333	1101111111111111011111111111111100001111111	L4	L4	L4
Problem panel	503	254222433224337262224433	1111111111111111111111111111111100001111111	L4	L4	L4
Problem panel	505	263623232443637272213523	1111111111111111111111111000000100001111111	L4	L4	L4
Problem panel	506	254434633544328254224533	1110000000000111110111001111111100001111111	No consensus	L4	L4
Problem panel	507	252333542134225252214233	1111111111111111111111100000000010001100011	No consensus	L4	L4
Problem panel	508	253734233435537363334523	1111111111111111111111111111110100001111111	No consensus	L4	L4
Problem panel	509	353655543236649452218423	1111111100011111100100001111111100001110111	No consensus	L4	L4
Problem panel	783_13	25456463324433(10)262326515	1111111111111111111111111111111100001111111	No consensus	No assignable	No assignable
Problem panel	1004_13	252333542134225252214233	1111111111111111111111100000000100001100011	No consensus	L4	L4
Problem panel	111_12	263623232445637252213523	1111111111111111111111111000000100001111111	L4	No assignable	L4
Problem panel	1131/13	253424533434339252214423	1110110111011111111111111111111100001101111	L4	L4	L4
Problem panel	1171/13	254542433224333262224413	1111111111111111111111111111111100001111111	L4	L4	L4
Problem panel	119/13	254524433444339263324533	1111111111111111111111111111111100001111111	No consensus	L4	L4
Problem panel	1223_13	244444633434246253214422	1111111111111111111111111111111100001111111	No consensus	No assignable	No assignable
Problem panel	1228_12	253720433046244453226655	1111110000001111111111111111111100001111111	L4	No assignable	No assignable

Problem panel	1287_12	554246433246436262326235	1111111111111111111111111111111100001111111	No consensus	No assignable	No assignable
Problem panel	1293_12	284446545432458250313423	0000000000000000000000000000000000001111111	No consensus	L2	L2
Problem panel	105	284446543442558252318423	0000000000000000000000000000000000111111111	L2	L2	L2
Problem panel	1372_12	253434233434737252314523	1111111111111111111111111111110100001111111	L4	No assignable	No assignable
Problem panel	1373_12	254444444443428252215323	1111110010001001000100000000000100000001100	No consensus	No assignable	No assignable
Problem panel	1376_12	254222433224339263224433	1111111111111111111111111111111100001111111	L4	No assignable	No assignable
Problem panel	159/12	263533343433534232313223	0000000000000000000000011111101000011111111	L4	L4	L4
Problem panel	279/13	234644533534223353314423	1111110110111111111101111101010100001100111	L4	L4	L4
Problem panel	335_12	443523344433334452314442	1111111111111111011111111111110100001111111	L4	L4	L4
Problem panel	397-12	234322342210323363214421	0111111100011111111100001111111100001111111	L4	L4	L4
Problem panel	404_13	234554633534335253224633	1111111111111111111111111000000000000000000	No consensus	L4	L4
Problem panel	413/13	251644522133439263214414	1111111111111111111100001111111100001111111	No consensus	L4	L4
Problem panel	502/12	263634533346339263224233	1111111111111111111111111000010100001111111	No consensus	L4	L4
Problem panel	543/12	222545145132336264234383	1101111111111111111100111111100001011111111	L1	L1	L1
Problem panel	555/13	265655542125238273314523	0010000100101111101000011101011100111111111	No consensus	L4	L4
Problem panel	559/13	3467363(11)2230(10)20460234542	1111111111111111110111111000000000000000000	No consensus	L4	L4
Problem panel	924_16	263422233433534253213223	0000000000000000000000010000001000011111111	L4	L4	L4
Problem panel	950-13	263434533434330353213423	1111110111111111110111111111110100001111111	L4	L4	L4

[a] loci order used: 580, 2996, 802, 960, 1644, 3192, 424, 577, 2165, 2401, 3690, 4156, 2163b, 1955, 4052, 154, 2531, 4348, 2059, 2687, 3007, 2347, 2461, 3171

[b] Using Tbminer platform

In MIRUtype column: "-" indicates non amplification; "_" indicates non amplification due to a deletion which eliminates the primer annealing region (Abascal E, Herrera DM, Herranz M, Santantón S, Martínez-Lirola M, et al. (2017) A deletion hampering appropriate typing of *Mycobacterium africanum*. Tuberculosis 103: 24-27)
